# Molecular Modeling-Guided Design of Phospholipid-Based Prodrugs

**DOI:** 10.3390/ijms20092210

**Published:** 2019-05-05

**Authors:** Milica Markovic, Shimon Ben-Shabat, Shahar Keinan, Aaron Aponick, Ellen M. Zimmermann, Arik Dahan

**Affiliations:** 1 Department of Clinical Pharmacology, School of Pharmacy, Faculty of Health Sciences, Ben-Gurion University of the Negev, Beer-Sheva 8410501, Israel; milica@post.bgu.ac.il (M.M.); sbs@bgu.ac.il (S.B.-S.); 2 Cloud Pharmaceuticals Inc., Durham, NC 27709, USA; skeinan@cloudpharmaceuticals.com; 3 Department of Chemistry, University of Florida, Gainesville, FL 32603, USA; aaron.aponick@gmail.com; 4 Department of Medicine, Division of Gastroenterology, University of Florida, Gainesville, FL 32608, USA; Ellen.Zimmermann@medicine.ufl.edu

**Keywords:** drug delivery, prodrugs, phospholipid, phospholipase A2, in-silico, molecular docking, molecular dynamics, molecular biopharmaceutics

## Abstract

The lipidic prodrug approach is an emerging field for improving a number of biopharmaceutical and drug delivery aspects. Owing to their structure and nature, phospholipid (PL)-based prodrugs may join endogenous lipid processing pathways, and hence significantly improve the pharmacokinetics and/or bioavailability of the drug. Additional advantages of this approach include drug targeting by enzyme-triggered drug release, blood–brain barrier permeability, lymphatic targeting, overcoming drug resistance, or enabling appropriate formulation. The PL-prodrug design includes various structural modalities-different conjugation strategies and/or the use of linkers between the PL and the drug moiety, which considerably influence the prodrug characteristics and the consequent effects. In this article, we describe how molecular modeling can guide the structural design of PL-based prodrugs. Computational simulations can predict the extent of phospholipase A_2_ (PLA_2_)-mediated activation, and facilitate prodrug development. Several computational methods have been used to facilitate the design of the pro-drugs, which will be reviewed here, including molecular docking, the free energy perturbation method, molecular dynamics simulations, and free density functional theory. Altogether, the studies described in this article indicate that computational simulation-guided PL-based prodrug molecular design correlates well with the experimental results, allowing for more mechanistic and less empirical development. In the future, the use of molecular modeling techniques to predict the activity of PL-prodrugs should be used earlier in the development process.

## 1. Introduction

Prodrugs are inactive drug derivatives that are enzymatically or chemically converted to the active drug moiety in-vivo [1,2]. The prodrug approach has become increasingly employed, aiming to improve the poor physicochemical drug properties and biopharmaceutical performance, in order to improve the effectiveness, allow for drug targeting, improve the drug safety profile, and facilitate formulation development and drug administration [3,4,5,6,7]. The prodrug design results in either a hydrophilic prodrug molecule, when the lipophilicity of the prodrug is lower than that of the parent drug, or a lipophilic prodrug with a higher lipophilicity than that of the parent drug [8,9,10]. Lipidic prodrugs contain the parent drug covalently bound to the lipid moiety-fatty acid, triglyceride, steroid, or phospholipid (PL). The use of the lipidic prodrug approach allows for incorporation into physiological lipid metabolic pathways, thereby bypassing absorption barriers, which are difficult to overcome otherwise [11].

PL-based prodrug design includes the covalent conjugation of the drug to the phosphate or glyceride backbone of the PL (*sn*-1 or *sn*-2 position) [12]. Different conjugation strategies lead to different fates of the prodrug within the body. Phospholipase A_2_ (PLA_2_) is the enzyme responsible for the hydrolysis of the *sn*-2 positioned fatty acid of the PL, and hence the conjugation of a drug moiety to this position may result in the enzymatic activation of the prodrug and the liberation of the free drug moiety [13]. However, if avoiding PLA_2_-mediated hydrolysis is aimed for, the drug needs to be conjugated to the phosphate group [14] or to the *sn*-1 [10] position of the PL. Careful PL-based prodrug design and structural considerations that could influence binding to the PLA_2_ active site can help to achieve the desired effects, such as site-specific targeting [15,16,17], lymphatic transport [18], and controlled release [19]. 

Computer-aided drug design (CADD) presents a computational simulation of the interactions that occur between the drug moiety and the protein (receptor and enzyme), while predicting the optimal drug molecule [20]. The main concern in using CADD is the inaccuracy of the simulation; indeed, oftentimes, the computationally obtained binding affinity between the drug and the protein does not correlate to the experimentally obtained data. Molecular docking is a fast routine method in the optimization of drug screening and design; however, the accuracy of such simulations is low (~20%) [20,21]. The reasons for this lack of precision are numerous, and include the use of a small database of molecules, the wrong choice of docking pose, the incorrect binding site of the target protein, a high docking score but unsuccessful molecular dynamics (MD) simulation, and more; this should be taken into account before performing molecular docking studies [22,23]. Physics-based computerized models, even though complex and time consuming, provide more accurate predictions, in comparison to empirical models such as docking (~80%). These include quantum mechanics/molecular mechanics (QM/MM) or free energy perturbation (FEP) methods [20]. Novel molecular modeling calculations, based on free energy perturbation can be employed to determine the affinity of the PLA_2_ enzyme towards PL-prodrugs [24,25]. In addition to this, simulations may provide information about the required prodrug structure modifications to optimize the enzyme activation, and by doing so, the experimentation time/burden can be reduced. 

This work will review the in-silico modeling techniques employed for the predictions of PL-prodrug activation. A brief overview of the PL processing pathways and novel utilizations of the PL-prodrugs will be provided, followed by a detailed description of the in-silico methods used for the prediction of the structure–activity relationship of PL-prodrugs, with a particular focus on the prerequisites for PLA_2_-mediated activation. The key advantages of molecular dynamics simulations for PL-prodrugs will be presented, as a useful tool in revealing structure-to-function relationship between macromolecules and for examining the conformational ensembles in a biorelevant surrounding.

## 2. Overview of Phospholipid-Based Prodrugs 

PLs found in the intestinal lumen may come from biliary or dietary sources, and are not absorbed intact. Before absorption, the enzyme PLA_2_ hydrolyzes the fatty acid in the *sn*-2 position of the PL, liberating free fatty acid and lysophospholipid, which are the absorbable lipolysis products [3,26,27]. Following absorption, the lysophospholipid and the fatty acid are reacylated to the PL, and this newly formed PL can now be incorporated into the surface of the lipoprotein within the enterocyte. Lipoproteins are released from the basolateral membrane of the enterocyte via exocytosis into the interstitial space, where they reach the lamina propria and undergo selective transport to the open capillaries of the mesenteric lymphatic vessels (lacteals), rather than to the blood vessels [3]. 

**Figure 1:**
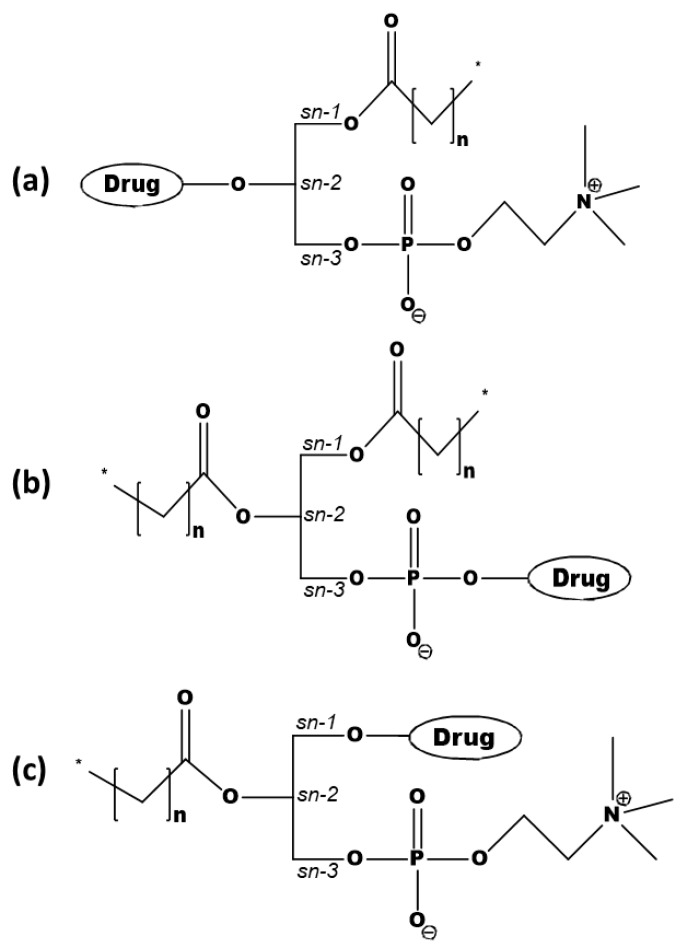
Prodrugs, with the active drug covalently attached to the (**a**) *sn*-2 position of the phospholipid (PL), (**b**) phosphate group, or (**c**) the *sn*-1 position of the PL.

The PL-prodrug design includes three different approaches—drug moiety that replaces the fatty acid attached to the PL glyceride backbone in the *sn*-2 (Figure 1a) position, drug moiety that is linked to the phosphate group of the PL (Figure 1b), or drug moiety in the *sn*-1 position of the PL glyceride backbone (Figure 1c) [12].

One of the main advantages of attaching the drug moiety to the phosphate group is avoiding hydrolysis by PLA_2_. Such a conjugation may provide an altered pharmacological effect, higher blood–brain barrier permeability, and may help in overcoming multidrug resistance (e.g., of nucleoside analogs) [28,29,30,31]. On the other hand, attaching the drug to the *sn*-2 position takes advantage of the PLA_2_-mediated activation, which results in the hydrolysis of the *sn*-2 fatty acyl bond and the liberation of the lysophospholipid and fatty acid [15,16,19,32,33,34]. Conjugation to the *sn*-2 position of the PL results in a PL-prodrug that has comparable surface properties and an aggregation performance like natural PLs. Secretory PLA_2_ (sPLA_2_) is overexpressed in numerous inflammatory [35,36,37] and malignant diseases [38,39], and this is where the PLA_2_-sensitive PL-prodrug approach is particularly useful. As PLA_2_ does not have a particular fatty acid selectivity, carefully designed PL-prodrugs can be susceptible to PLA_2_ hydrolysis, and can release the active drug moiety at the specific site of the enzyme overexpression. 

PL-prodrugs can also be directed into the intestinal lymphatic system, depending on their structure. In such cases, it is crucial to determine whether the polar part of the PL-based prodrug (i.e., active drug moiety) could influence the ability of the prodrug to stabilize the lipoproteins created within the enterocyte [3]. For example, following the oral administration of PL- fluorouridine, the prodrug is absorbed into the enterocyte via a deacylation–reacylation cycle (PL uptake route), and conveyed into the intestinal lymphatic system [40]. In another example, it was shown that the PL-prodrug with a direct conjugation between PL and the active drug moiety in the *sn*-2 position is directly absorbed by the enterocytes, and enters the lymphatic system intact [18]. The general prerequisite for intestinal lymphatic transport is that the compound has log *p*-value above 5, and solubility in TG of >50 mg/g [41]. The advantage of lymphatic transport is that lipophilic compounds can bypass the first-pass hepatic metabolism and become orally bioavailable; which may allow for control of the drug delivery rate to the circulation, and for controlled drug delivery. 

The conjugation of the drug moiety to the *sn*-1 position of the PL has been employed for anticancer ether lipids (ProAELs), with promising results [42,43]. In addition to this, ProAELs containing a thio–ester bond in the *sn*-2 position were synthesized and analyzed as well. The PLA_2_-mediated activation of the newly formed thio–ester pro anticancer ether lipid (S-ProAEL) was shown to be decreased when compared to the ProAELs with an ester bond in the *sn*-2 position, which was explained by the MD simulations, described hereinafter [44].

## 3. Computational Optimization of PL-Prodrug Design

Different molecular modeling methods are in use for determining the activity of PLA_2_ towards PL-prodrugs (molecular docking and MD simulations). These simulations can point out the structural adjustments of the PL-drug conjugates required to obtain the highest degree of prodrug activation. The molecular docking study of the complex characterization between the PL and the drug is presented in Section 3.1. Oftentimes, the PL-drug conjugates are designed to target an enzyme PLA_2_, and, in such cases, the drug design and usage of the linker/spacers between the PL and the drug is employed. In such cases, molecular docking simulations are more challenging for distinguishing between the activation of various PL-drug conjugates.

A frequently used technique is molecular dynamics (MD) simulation, a well-known method for obtaining information about the structure-to-function relationship of macromolecules [45]. The activity of PLA_2_ towards PLs relies on several aspects, such as the cell membrane properties, formation of the Michaelis–Menten complex, entrance of water molecule to the enzyme active site, steric hindrance between the PL-prodrug, and the enzyme. 

Three main MD approaches were used for the optimization of the PL-drug conjugates, and will be covered hereinafter. First, the use of the free energy perturbation method using thermodynamic integration and umbrellas sampling/the weighted histogram analysis method (WHAM) method for determining the relative binding free energies of PL-drug conjugates with different linker lengths will be described (Section 3.2). An additional approach for determining the structural prerequisite of the PL prodrug for PLA_2_ activation, based on the stabilization of the Michaelis–Menten complex and the availability of a water molecule in the catalytic cleft (to act as a nucleophile) will be described in Section 3.3. Lastly, the influence of side groups in the PL-drug conjugate design analyzed using MD simulations and density functional theory will be covered (Section 3.4).

### 3.1. Molecular Docking

The use of molecular docking to guide PL-based prodrugs is rather rare, and in fact, only couple studies to date report such attempts. Molecular docking studies were employed to confirm if the drug rosuvastatin is able to form a complex with the PL analogue (phosphatidylcholine transfer protein) [46]. The docking studies were performed using Schrodinger software version Glide 5.5., and the drug–protein interactions were studied using PyMol software (M/s Schrodinger LLC, New York, NY, USA). The studies showed the site(s) of interaction between the drug and protein. They interact through weak intermolecular H-bonds between the free hydroxyl group of the drug with the proteins’ quaternary amine group. This leads to the minimization of the total free energy for obtaining conformation that is thermodynamically stable. Thus, the docking studies established that rosuvastatin is able to form a complex with the phosphatidylcholine transfer protein, probably because of the similar physiochemical properties to that of PL [46]. The molecular docking was used to understand the structural restrictions of the *sn*-1 substitutioned PL-prodrugs; however, the results of the simple docking were not conclusive, and demonstrated that all *sn*-1 substitutioned PL-prodrugs fit to the PLA_2_ binding cleft [47]. Overall, it appears that a significant advancement of the field is needed before successful molecular docking can be achieved; combining molecular docking with other modeling methods may result in better outcomes than the use of docking alone.

### 3.2. Free Energy Perturbation (FEP) and Molecular Dynamics (MD) Simulation: Linker Length Dependent PLA_2_-Mediated Activation

**Figure 2:**
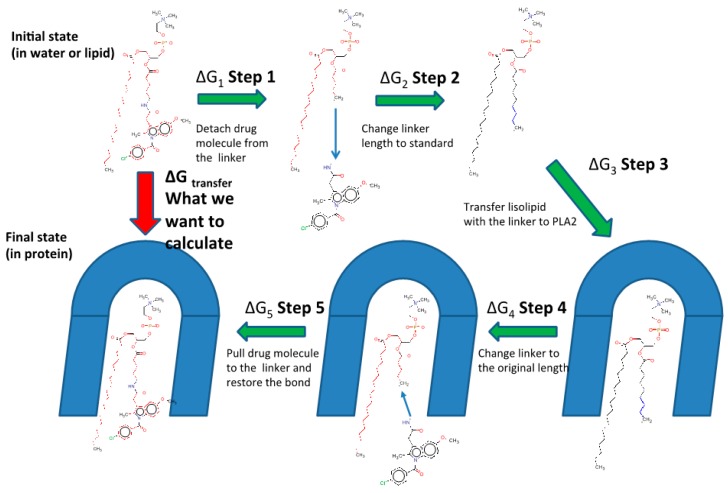
Alchemical cycle to compute free energy of the transfer of the PL-indomethacin prodrug molecules to the PLA_2_ transition state geometry. (ΔG_1_ + ΔG_5_), the energies for bond cutting/forming cancel out. Free energies of pulling the drug molecule to the end of the linker are computed using the umbrella sampling technique. (ΔG_2_ + ΔG_4_), the differences in free energies of changing the linker length in the complex with PLA_2_ and in the initial state (lipid or water) are computed using the free energy perturbation (FEP) method.

An MD combined with the free energy perturbation approach was previously developed by us, in order to determine the relative activation of the PL-prodrug containing different linker lengths between the PL and the drug within the enzyme PLA_2_ [24,25]. The overall aim was to use PLA_2_ to hydrolyze the *sn*-2 acyl bond of a PL-drug conjugate; this way, the drug moiety would be released specifically at the site of action, where the enzyme is overexpressed [16,19,24,25]. However, for the PLA_2_-mediated activation to occur, the prodrug molecule should adopt a well-defined transition state geometry in the PLA_2_ active site. This transition-state geometry is characterized by strong interactions of the *sn*-2 carbonyl oxygen with the calcium atom (ion located in the PLA_2_ active site), as well as the specific position of the protein His residue, which activates a water molecule for the nucleophilic attack on the prodrug acyl bond. The prodrug concentration in the transition state geometry with PLA_2_ and the rate of the cleavage reaction are determined by the binding free energy of the prodrug in the enzyme active site. Lowering the binding free energy correlates with a higher degree of binding between the PL-drug conjugate and PLA_2_. The PL-prodrugs of diclofenac and indomethacin with different linker lengths in the *sn*-2 position of the PL were designed and synthesized; the differences in the PLA_2_ transition state binding energies of the prodrugs were calculated while decreasing/increasing the linker length. We analyzed the changes in the rates of the PLA_2_-mediated prodrug hydrolysis by calculating the relative binding energies through computing the free energy changes that occur when the linker length is changed. Two states are compared-the initial state (free prodrug molecules in solvent or lipid phase) and final state (prodrug with PLA_2_ in the transition state geometry). Both human and bee venom PLA_2_ enzymes were simulated, aiming to determine the relevance of extrapolation from the bee venom PLA_2_ to the human enzyme. The enzymes were modeled with the AMBER ff-03 force field and the TIP3P model described water molecules. A GAFF force field with AM1-bcc partial atomic charges was set for the PL-drug conjugates. The linker length is changed in each state using alchemical transformation (i.e., adding or removing atoms on the computer), and the changes in the free energy are calculated using a thermodynamic cycle (Figure 2). To compute the free energy of the transfer of a prodrug molecule from its initial state (water/lipid) to its final state (transition state complex of PLA_2_), we analyzed the following five steps: (1) at initial state, a bond connecting the drug moiety to the PL is cut and the drug is pulled away from the lysophospholipid-linker complex (step 1); (2) the linker length is changed to standard (step 2); (3) transfer of the lysophospholipid with a standard linker length from the initial state to transition state of PLA_2_, where the free energy of this step is the same for all of the prodrug derivatives (step 3); (4) the prodrug molecule is in the final state (PLA_2_), and the linker length is changed from standard to the initial length (step 4); and (5) the drug molecule is pulled towards the end of the linker and the bond is created (step 5). The sum of the free energies from the steps 1–5 results in the free energy of the PL-prodrug molecule transfer to the PLA_2_ transition state. The relative binding free energies of the PL-drug conjugates in the transition state complex of the PLA_2_ enzyme ($\Delta \Delta G^{tr}$) are calculated as the difference between the final state free energy (conjugate in PLA_2_, $\Delta G_{mod}^{f}$) and the initial state free energy (conjugate in water/lipid, $\Delta G_{mod}^{i}$). $\Delta G_{1}^{tr}$ and $\Delta G_{2}^{tr}$ represent the free energies of adding/removing -CH_2_ units in the final and initial state, respectively; $\Delta \Delta G^{tr}$ is calculated from the following equation:

$$\Delta \Delta G^{tr}=\;\Delta G_{2}^{tr}-\;\Delta G_{1}^{tr}=\;\Delta G_{mod}^{f}-\;\Delta G_{mod}^{i}$$

**Figure 3:**
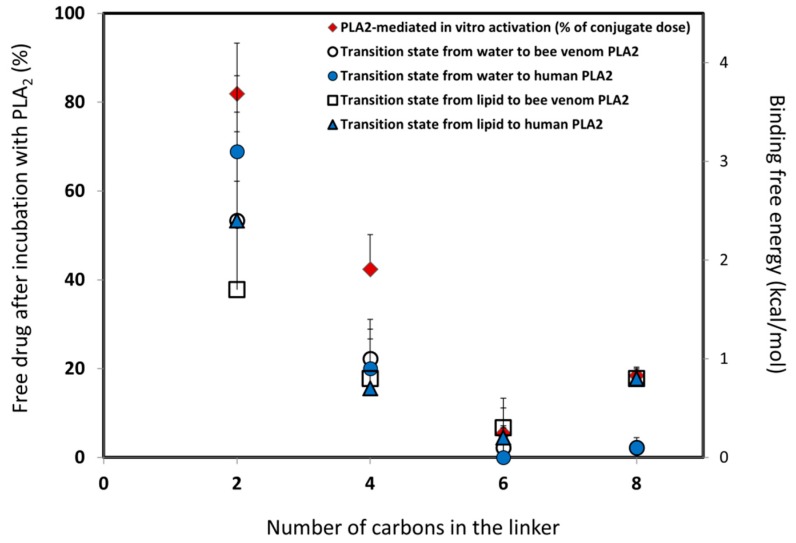
In-silico/in-vitro correlation for PL-diclofenac prodrugs: in-silico prodrug binding free energies in PLA_2_ transition state from the initial state of water or lipid (kcal/mol) versus in-vitro results for conjugates with a linker length of two-, four-, six-, and eight-carbon atoms (% of intact complex). Reproduced with permission from the authors of [25].

The free energy of removing the linker -CH_2_ units were computed using the thermodynamical integration (TI) method, and by keeping the distance between the end of the drug moiety and the new linker end constrained [48]. Thermodynamic integration (TI) computes the difference in free energy between two given states by ensemble-averaging the enthalpy changes along the path connecting two states. The free energy associated with attaching the drug moiety to the shorter linker was computed using the umbrella sampling (US) and weighted histogram analysis methods (WHAM), by progressively applying different harmonic constraints between the linker end and the drug moiety [49]. The umbrella sampling (US) method involves applying set of harmonic constraints between the atoms in the system, and observing the changes in the average distances between the constrained atoms. The series of US simulations were analyzed using the WHAM method. The in-silico results obtained in this way for both the PL-diclofenac and PL-indomethacin prodrugs demonstrated an excellent correlation with the experimental results [24,25]. Lower rates of PLA_2_-mediated hydrolysis from the experimental study were found to be proportional to the higher binding free energies in the PLA_2_ transition state geometry. In the case of the indomethacin prodrugs, the PL-conjugate with a linker length of 5-CH_2_ units was shown as the optimal for activation with PLA_2_ (with the lowest energy for activation), where the linker lengths beyond 5-CH_2_ units failed to further improve the activation rate. The PL-diclofenac prodrugs demonstrated a gradual decrease in the relative binding energy from two- to six-carbon atoms, with the highest rate of activation for six -CH_2_ units, whereas the linker lengths beyond this point (towards C8 linker) showed an increase in the binding energy. The correlation between in-vitro and in-silico results for PL-diclofenac prodrugs is demonstrated in Figure 3. 

**Figure 4:**
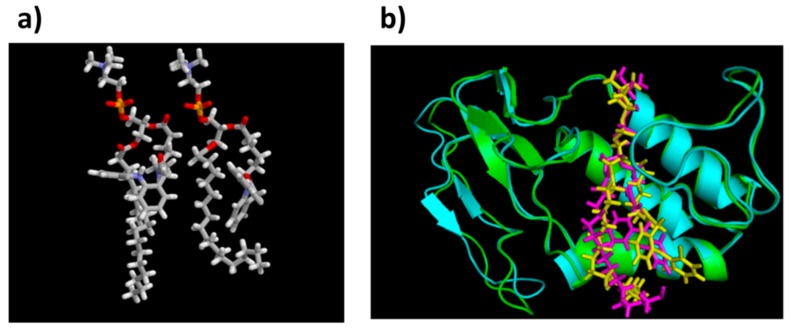
(**a**) Optimized transition state geometries of PL-diclofenac prodrugs with 5 vs. 6 carbons linker, within human PLA_2_ (the protein structure was removed for simplicity); (**b**) Equilibrated structures of PL-diclofenac conjugates with linker lengths of 2- and 6- CH_2_ units in bee venom PLA_2_. Figure 4b is reproduced with permission from [25].

Optimized structures for the PL-diclofenac prodrugs with a linker length of 5- and 6-carbon atoms, and equilibrated structures of PL-diclofenac prodrugs within PLA_2_ are presented in Figure 4a,b, respectively.

Computerized methods to predict the binding affinities utilizing physics-based models such as FEP are very time and compute thoroughgoing, but far more accurate (approximately 80%) in comparison to empirical methods such as docking (20%) [20]. Indeed, it can be seen in the research presented in this section that the FEP simulations were in excellent correlation to in-vitro results. The FEP method is highly accurate, however, it is very time and compute intensive.

### 3.3. MD Simulation: Michaelis–Menten Complex and Water Availability in PLA_2_-Mediated Activation 

**Figure 5:**
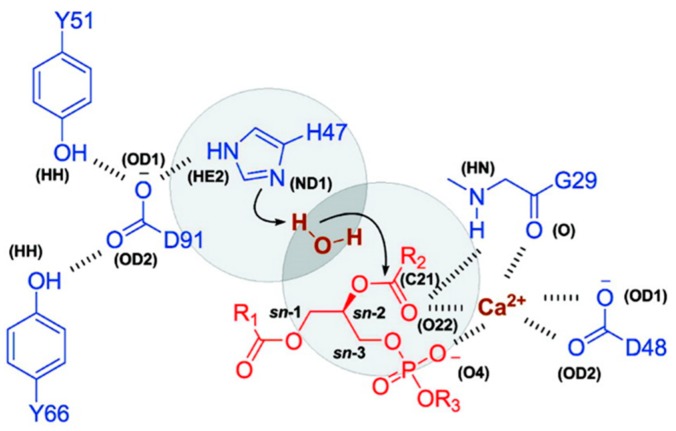
Site of PLA_2_ with protein residues (blue), substrate (red), calcium, and water (brown). The H–S region is represented in circles. Types of atoms in brackets are taken from the Protein Data Bank. Reproduced with permission from the authors of [50].

One of the prerequisites for PL-prodrug activation (PLA_2_-mediated hydrolysis) are a stable Michaelis–Menten complex and a molecule of water (a nucleophile that is free to enter the catalytic site of PLA_2_). Novel drug delivery systems containing PLA_2_-sensitive PL-drug conjugates were produced, in which the lipophilic anticancer drugs (such as retinoic acid-ATRA) are directly attached to the *sn*-2 position of the phospholipids [50]. Experimental studies showed that the desired delivery profile of the free ATRA was not feasible because of a lack of PLA_2_-substrates affinity. MD simulations were performed in order to understand why a direct conjugation between the *sn*-2 position of the PL and ATRA lacks PLA_2_-mediated activation, and which modifications are required in the structure of the PL-prodrug to regain this activation [15,32,50]. PL-prodrugs were overlapped with the pre-existing substrate, which was consequently deleted. All of the simulations were performed using NAMD software (Theoretical and Computational Biophysics Group, Urbana, IL, USA) with the Charmm27 all-atom parameter set and TIP3 water model. The initial MD simulations were performed in 100 ps and heated to 300 K. Each simulation was conducted for 10 ns in a constant number of atoms, pressure, and temperature [50]. The direct conjugation between ATRA and PL did not achieve a stable Michaelis–Menten complex, which was shown in the following experimental conditions: no PLA_2_-mediated hydrolysis was observed [15,32]. Interestingly, these results were in excellent agreement with previous studies of PL–valproic acid conjugates, in which direct conjugation resulted in no affinity to the enzyme [18]. For this reason, a six-carbonic linker was introduced between the PL and ATRA, for which MD simulations demonstrated a stable Michaelis–Menten complex. Further simulations tested the ability of the water molecule to enter the catalytical site of the enzyme [50]. The catalytical site of PLA_2_ contains an aspartic acid–histidine dyad, a calcium-binding site, and a water molecule (Figure 5). The water molecule acts as a nucleophile and should enter the region in-between H47^ND1^ and S^C21^ (H–S region), as demonstrated in Figure 5. The relative water count from the simulation of the PL-ATRA prodrug with a six-carbon linker were comparable to that of natural substrate (1,2-dipalmitoyl-*sn*-glycero-2-phosphoglycerol); the water count for the conjugate that includes the direct conjugation was found to be very low. The PLA_2_-mediated hydrolysis in-vitro was in correlation with the MD simulations. The rationale for this phenomenon is the rigid molecular structure of the ATRA (a methyl group in the vicinity of the carboxyl group); in contrast, the physiologically-occurring, saturated fatty acids have a flexible structure with no branching. 

This computational approach offers a way to determine which structural modifications can allow for an effective liposomal enzyme-triggered release, even for sterically hindered substrates. 

### 3.4. MD Simulation and Density Functional Theory: Side Group Influence, S-Ester vs. O-Ester

Biophysical techniques and MD simulations were also used to understand, optimize the design of, and reveal the structural restrictions of the substitution on the *sn*-1 PL-prodrugs [44,47]. The initial study included the molecular docking of the PL-conjugates to PLA_2_. However, the results of the simple docking demonstrated that all PL-conjugates fit the binding site of the enzyme [47]. Consequently, the MD simulation was conducted to study PL-prodrugs with *sn*-1 positioned groups of different sizes and/or polarity on a molecular level, in order to reveal the structural restrictions that would present an obstacle for PLA_2_ enzymatic activation. The MD simulation was conducted using the software NAMD (Theoretical and Computational Biophysics Group, Urbana, IL, USA), with the Charmm27 all-hydrogens parameter set, and using the TIP3 water model. The system was heated to 300 K after 100 ps. Every simulation lasted 10 ns in the NPT ensemble; through all of the simulations, a time step of 1 fs was used [47]. It was concluded that the PL-conjugates with a branching substitute group in the *sn*-1 position block the water molecule from entering the catalytic site, and hence would restrict hydrolysis; these results were also confirmed experimentally [47]. 

**Figure 6:**
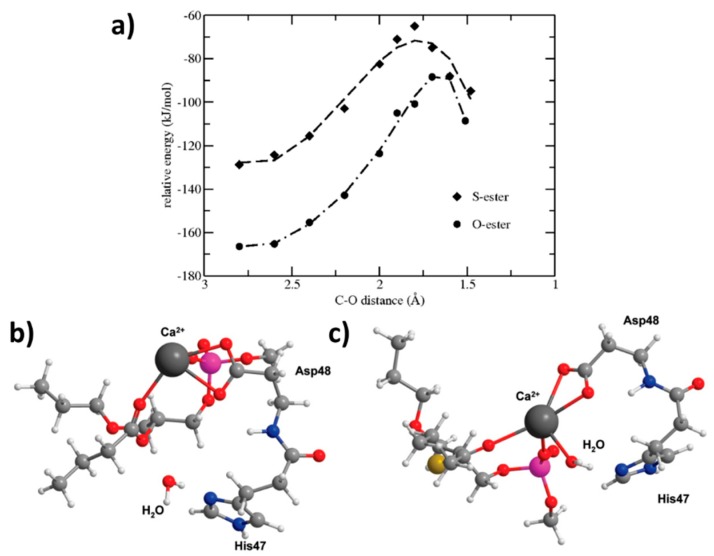
Energies for the O-ester and S-ester substrates calculated with different, fixed distances between the oxygen from water and the carbonyl carbon. Conformations of these minimized ester substrates are considerably different; (b) O-ester with the associated water molecule close to the carbonyl group; (c) the S-ester prefers a different location in the active site with a large distance between the water molecule and the carbonyl group. Ca^2+^ (dark gray), P (pink), O (red), N (blue), S (yellow), C (light gray) and H (white). Reproduced with permission from [44].

An additional study on a thio-ester pro anticancer ether lipid (S-ProAEL) provided the mechanisms behind the PLA_2_-mediated activation of the thio–esters, using the same software and similar MD simulation parameters, as the previous study. The in-vitro PLA_2_-mediated rate of the hydrolysis was found to be slower for the cytotoxic S-ProAEL when compared to the natural substrate. The MD simulation and density functional theory (DFT) were carried out in order to study the S-ProAEL-PLA_2_ interactions, as well as the water availability at the catalytical site, and energy of the transition state formation, respectively [44]. The thio–esters (S-ProAEL) were compared with a natural substrate (O-ester). Both the S-ProAEL and natural substrate demonstrated a perfect fit into the binding pocket of the enzyme. A stable Michaelis–Menten complex and comparable dynamics of PLA_2_-prodrug complex were achieved. The availability of the water molecule that plays an important role in the activation of various derivatives of PL-prodrugs was found to be optimal for the thio–ester as well. Further studies were conducted to elucidate the difference between the hydrolysis rates of the natural O-ester and S-ester. The two esters have considerably different conformations (Figure 6b,c), and as a result, a much longer distance among the carbonyl and the water molecule in the S-ester in comparison to the O-ester. The DFT calculations (B3LYP/LACVP) revealed a similar hydrolysis mechanism when compared to natural substrate; however, the energy of the activation was shown to be considerably higher (Figure 6a). Therefore, the in-vitro variation in the hydrolysis rate of the O- and S-ester can be explained by the innate electronic difference between sulfur and oxygen [44]. DFT studies require extensive screening and time-consuming MD simulations; however, this example highlights the importance of combining MD and DFT methods in order to elucidate the different binding affinities towards the PLA_2_ active site.

The examples described above demonstrate that the computational simulations of the PL-based prodrugs may support the experimentally obtained results. In the future, the use of molecular modeling techniques to predict the activity of PL-prodrugs should be used to a greater extent, prior to the prodrug design itself. Elucidating the active site and the mechanism of the PLA_2_ action towards PL-drug conjugates allows for the optimized design and synthesis of prodrugs with tunable hydrolysis rates. 

## 4. Discussion

In order to create an optimal prodrug candidate, a number of empirical steps are employed, making the development process effort-, time-, and money-consuming. This is where modern computational techniques can be used to produce reliable predictions, in order to reduce the number of syntheses and/or experiments required to achieve an optimal design and appropriate activation. Molecular docking presents the target-based drug design, that is, ligand binding to its receptor, target protein [51]. However, in the case of PL-drug conjugates, this approach was not optimal; numerous structural and binding site parameters need to be taken into account, and several different techniques and approaches were employed instead. A significant advancement of the field is needed before successful molecular docking can be achieved; we posit that combining the molecular docking with the other modeling methods may result in better outcomes than the use of docking only. The MD simulations are biologically relevant, and the data about the macromolecules obtained in this way can move the usual paradigm of studying single structures to evaluate conformational ensembles; it is extensively employed, and the simulation times are very fast (close to milliseconds). However, some obstacles remain, including a lack of optimized tools and representation standards [45]. 

**Figure 7:**
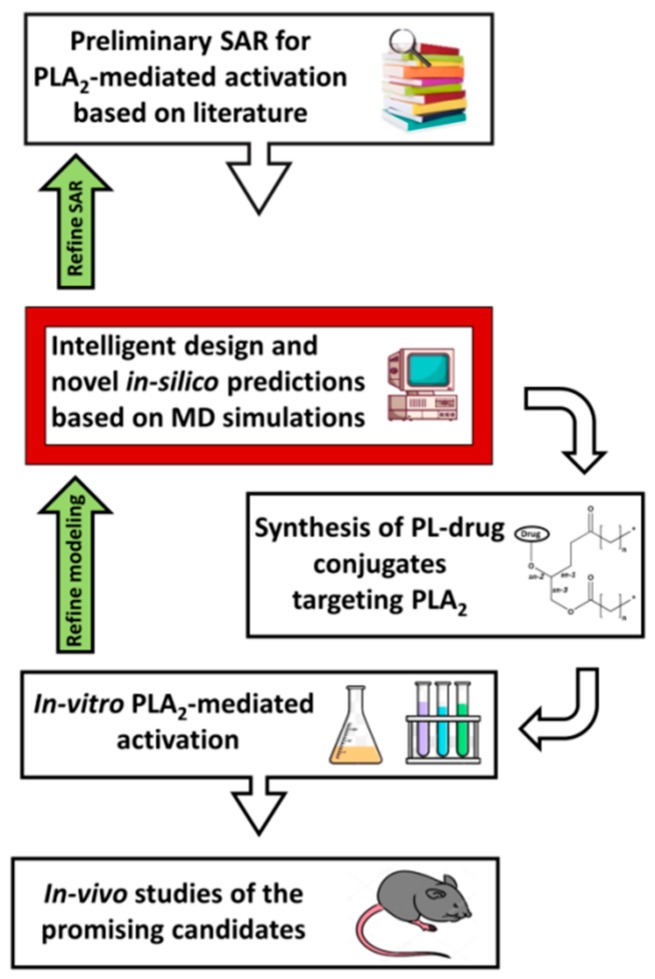
Flow-chart of our proposed PL-drug conjugates development process. SAR—structure-activity relationship; MD—molecular dynamics; PL—phospholipid; PLA_2_—phospholipase A_2_.

FEP can predict the affinity differences for congeneric ligands with a high level of accuracy and very high computational times. Calculating the relative binding free energies is less computationally intensive than absolute binding free energy calculations, and points directly onto the hit-to-lead and lead optimization processes; using such predictions between a reference molecule and new ones can be used to prioritize molecules for synthesis [52]. Density functional theory (DFT) allows for studying individual molecules directly, and together with the growing computational tools, the methods based on the DFT can be applied to larger and larger systems. Nevertheless, for an accurate DFT study, it is required to have a high throughput screening, conformational searching, and long timescale MD simulations. As demonstrated in Section 3, a combination of physical methods and computational simulations provides more accurate and biologically relevant results. A diagram of our proposed PL-prodrug development process in presented in Figure 7; the molecular modeling techniques described in this work can lead the optimal structural design of PL-based prodrugs, anticipated to be activated by the enzyme PLA_2_, and thereby achieve its desired characteristics.

In the PL-prodrug design, various conjugation approaches are employed, as well as linkers between the PL and the drug moiety. These structural modifications can significantly influence the prodrug properties and its consequent effects, such as the drug release from the prodrug complex [19], overcoming drug resistance [29,30], or drug targeting [16,32,34]. In cases like this, the use of molecular modeling is particularly useful, and can provide information about optimal modification in the structure of the linker/bond needed for the suitable activation of the enzyme. Various molecular modeling techniques aid different aspects of PL-prodrug optimization and design; molecular docking studies were able to reveal the site(s) of interaction between the drug and protein, whereas the FEP and MD simulations can aid in the design of the linker length between the PL and the drug in order to optimize PLA_2_ activation, and DFT theory can reveal changes in the electronic difference between the atoms making up the bond between the PL backbone and the drug.

In the case of the PL-diclofenac and PL-indomethacin prodrugs, the computational method permits the optimization of the chemical structure of the molecular linker connecting the drug moiety to the PL, and reducing the amount of chemical synthesis needed for developing the effective PL-prodrugs. These methods were shown to be in excellent correlation with the in-vitro data (Figure 3). It was clearly shown that the linker design has a crucial role in the degree of activation by PLA_2_, and the degree of the active parent drug released at the site of action. However, the optimal linker length needs to be determined on a case-by-case basis, as the volume and size of the drug itself highly influences the steric interactions. Additionally, both bee venom and human PLA_2_ isoforms resulted in comparable rates of activation, indicating that the bee venom PLA_2_ may be used as a substitute for the human enzyme in experimental conditions [24,25]. MD simulation of retinoic acid also demonstrated a high correlation with the experimentally obtained result; the incorporation of a 6-carbon linker allowed for the ATRA to overcome the lack of PLA_2_ hydrolysis, which originated from its rigid structure. This simple modification allowed for the PL-ATRA prodrug to be incorporated into the liposomes, and proved that even the sterically hindered retinoid drugs can be incorporated into formulations and achieve enzyme-triggered drug release in the cancer treatment [50].

The MD calculations on an atomic level also revealed that the ProAELs with branching linkers located at the first carbon atom of the *sn*-1 substitute or at the ester group were shown to block the water molecule from the catalytical place in the PLA_2_ active site. The results were in correlation with the in-vitro results [47]. If, however the modification is made from the ester to thio group in the *sn*-1 position, the variability in the PLA_2_-mediated activation cannot be explained by the lack of water molecules available at the catalytical site, but rather, further studies into the energetic changes of the enzymatic reaction are required (Figure 6a). In addition to this, the PLA_2_ is evolutionary optimized to catalyze only the O-esters, and does not activate the unnatural S-esters equally well [44].

Computational methods that are based on docking and molecular mechanics encounter obstacles when it comes to predictions of transient states in enzymatic reactions; this is when the simulations based on quantum methods, such as DFT, are employed. Many conjugation strategies and the use of a linker (linear or branched) between the PL and the drug moiety may be used in the PL-prodrug design. This modification can significantly influence the PL-prodrug features, including the PLA_2_-mediated activation, GI stability, and/or drug release from the prodrug complex. The design of linkers/spacers with variable release profiles, which would give rise to the development of optimal PL-prodrug candidates, is a significant future research direction.

Using these complex in-silico calculations may increase the success rate and can lower the overall costs of PL-prodrug development, making way for faster and more accurate prodrug discovery, design, targeting, and release.

## 5. Conclusions

Novel molecular modeling calculations are employed to determine the affinity of the PLA_2_ enzyme towards PL-prodrugs, and to provide information about the required prodrug structure modifications in order to accomplish optimal enzyme activation; the MD simulations were proven to be a very useful tool to achieve this aim. As molecular docking and molecular modeling techniques encounter many obstacles in predictions, a combination of physical methods (DFT) and computational simulations (FEP) provide more accurate and biologically relevant results. However, the optimal structure needs to be determined on a case-by-case basis, as the volume and size of the drug itself highly influences the steric interactions with the active site of the enzyme. In the future, the use of molecular modeling techniques to predict the structure-to-activity relationship of PL-prodrugs should be employed prior to the prodrug synthesis-linker lengths between the PL and the drug should be optimized in order to obtain the most extensive activation by the PLA_2_ enzyme.
